# Versatility of live-attenuated measles viruses as platform technology for recombinant vaccines

**DOI:** 10.1038/s41541-022-00543-4

**Published:** 2022-10-15

**Authors:** Aileen Ebenig, Mona V. Lange, Michael D. Mühlebach

**Affiliations:** Division of Veterinary Medicine, Paul-Ehrlich-Institut, D-63225 Langen, Germany

**Keywords:** Infectious diseases, Drug development, Live attenuated vaccines

## Abstract

Live-attenuated measles virus (MeV) has been extraordinarily effective in preventing measles infections and their often deadly sequelae, accompanied by remarkable safety and stability since their first licensing in 1963. The advent of recombinant DNA technologies, combined with systems to generate infectious negative-strand RNA viruses on the basis of viral genomes encoded on plasmid DNA in the 1990s, paved the way to generate recombinant, vaccine strain-derived MeVs. These live-attenuated vaccine constructs can encode and express additional foreign antigens during transient virus replication following immunization. Effective humoral and cellular immune responses are induced not only against the MeV vector, but also against the foreign antigen cargo in immunized individuals, which can protect against the associated pathogen. This review aims to present an overview of the versatility of this vaccine vector as platform technology to target various diseases, as well as current research and developmental stages, with one vaccine candidate ready to enter phase III clinical trials to gain marketing authorization, MV-CHIK.

## Introduction

Measles has been known for centuries as a scourge of humanity, killing millions of children in historical times per annum^1^. With its introduction into the human population dating back to the sixth century BCE^2^, the death toll over time has been immense. The causative agent, the measles virus (MeV), was isolated in 1954 from a small boy with acute measles, David Edmonston. This isolated virus was passaged in the laboratory on human and animal primary cells, as well as on immortalized cell lines^3^ that retrospectively do not express one of the two physiologically relevant human cellular entry receptors SLAM F1^4^ or nectin-4^5^. By repeated passaging (at least 80 times), MeV adapted and became attenuated^6^ through accumulation of many mutations throughout its genome^7^. Identification of discrete genetic determinants of attenuation was not successful, indicating that multiple mutations were responsible for the attenuated phenotype^8–10^. Most obvious has been the change of entry receptor tropism: Pathogenic patient isolates and virus strains use only signaling lymphocyte activation molecule (SLAM F1, also known as CD150)^4^ on activated immune cells and nectin-4 as host exit receptor on the basolateral side of tracheal epithelial cells^5^. Virus strains passaged in tissue culture adapt by additionally using the ubiquitous surface molecule CD46 for cell entry^11,12^ by acquiring as few as four amino acid substitutions in the hemagglutinin (H) glycoprotein^13^.

These adapted, live-attenuated viruses are no longer pathogenic, but still replicate in vitro and in vivo, infecting the same host cells and tissues in vivo as their pathogenic ancestors despite theoretically expanded entry receptor tropism^14^. In any case, the attenuation of live-attenuated vaccine-strain MeV is extremely robust: reversions to virulence have not been described, and only severely immunocompromised vaccinees are excluded from vaccination due to a greatly enhanced chance of experiencing severe side-effects of the measles vaccine. Otherwise, only pregnant women are excluded for theoretical reasons as well as persons allergic to vaccine components^15^. In fact, even measles vaccination of HIV-1 infected patients is recommended unless their CD4^+^ T cell count is below 200 cells/μl, in that there must be at least some residual T helper cell activity^16^. This is in accordance with an extraordinary safety profile; vaccination with the combined MMR vaccine (immunizing in addition against mumps and rubella) is only rarely associated with severe adverse events^17^. On the other hand, efficacy is high, with a protection rate against the measles of 93% after one vaccination^18^. Moreover, the longevity of protection after natural measles infection, which usually results in life-long immunity, seems to be fostered at least partially also by the measles vaccine^19^. Usually, one successful vaccination against MeV protects for life with low secondary vaccine failure rates in the range of less than 0.2%^20^, although it remains to be elucidated if frequent contact to circulating wild-type MeV had a boosting effect on vaccine-primed immunity in the past. An indication for such an effect may be seen in some progressive decrease of protection afforded by the MeV vaccine over the last two decades in the absence of endemic circulation of wild-type (wt) virus^21^.

With the advent of recombinant DNA technologies, it became feasible to manipulate cloned virus genomes. Problems specific to the biology of MeV, and other members of the order of *Mononegavirales*, took another 20 years to be resolved, until it became feasible to generate recombinant MeV from manipulated plasmid DNA. *Mononegavirales* carry a single-stranded viral RNA genome of negative-strand polarity, which cannot be used as a transcriptional template to establish viral replication. Instead, the virus replication machinery, the ribonucleoprotein complex (RNP), consisting of the viral RNA genome, the RNA-dependent RNA polymerase (L) protein, the polymerase co-factor phosphoprotein (P), and the nucleocapsid protein (N), must assemble to generate replication centers. Thereby, the RNA genome or antigenome of MeV entirely covered by N homopolymer is the template for the polymerase complex of L and P. Since one N protein covers six nucleotides of the genome, MeV genomes have to obey the so-called “rule-of-six”, i.e. total number of nucleotides has to be a multiple of six, and no other multiple of nucleotide deletions or additions are tolerated. These complexes of viral proteins and RNA replicate the viral genome in the cytoplasm of infected cells, and transcribe all viral mRNAs from single genes encoded by the viral genome. These viral mRNAs are translated by the cellular protein biosynthesis machinery, and the resulting proteins and the replicated viral genome assemble into infectious daughter viruses; particles budding from the cellular membrane to generate enveloped, pleomorphic particles^22^.

Initially established for rabies virus^23^, reverse genetics methods to “rescue” recombinant *Mononegavirales* were first demonstrated for MeV in 1995 by the group of Martin Billeter^24^. The technical details of this and other rescue systems, which were later developed to enhance efficacy, are not in the focus of this review and have been described elsewhere^25^.

**Fig. 1:**
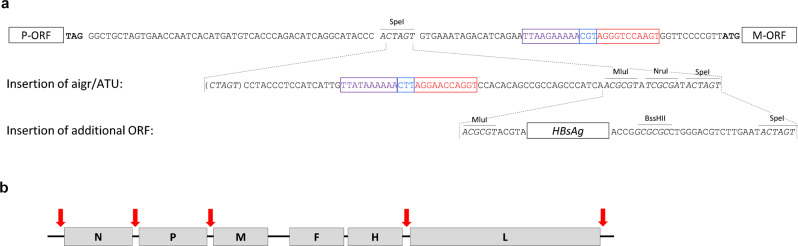
Strategy to insert additional genes into MeV genomes. **a** Schematic depiction of the DNA Sequence of the intergenic region between P and M genes of MV_NSe_ revealing insertion of an additional intergenic region (aigr) to be utilized as an additional transcription unit (ATU) to encode extra genes in the genome of recombinant MeV, in this example the HBsAg^30^. Shown is the sense strand of DNA sequences used for cloning. Open reading frames (ORFs) are depicted by black boxes, recognition sequences for restriction endonucleases are outlined in sequences in italics^30^ and respective endonucleases are indicated. Conserved transcription termination (lilac) and re-initiation (red) sequences of the MeV polymerase separated by the non-transcribed intergenic triplet CT/GT (blue) are color coded and framed. Bold, stop and start-codons for translation of flanking viral P and M protein ORFs. **b** Schematic depiction of rec. MeV genomes. Gray boxes indicated MeV ORFs, red arrows positions where ATUs have been inserted and used for the expression of additional transgenes.

After the generation of recombinant MeV became feasible, strategies were then developed to encode extra proteins in the cloned viral genome. This process is straightforward, since the genome of *Mononegavirales* is organized in gene cassettes. Conserved sequences in the intergenic regions separating the single gene cassettes ensure expression of the respective genes’ mRNA. These sequences cause the viral polymerase complex, which attaches to the RNA genome only in promoters located in the proximal leader and trailer regions of the genome, to terminate transcription of mRNA at the end of the upstream transcription unit and to re-initiate transcription for the downstream unit^26^. By duplication of the termination/re-initiation sequences in an intergenic region^27^, an additional transcription unit (ATU) can be generated (Fig. 1).

If a foreign gene is inserted into such an ATU, it is transcribed by the viral polymerase complex and is then translated in infected cells alongside viral genetic components. Since the viral polymerase complex can only attach to the MeV genome in the terminal promoter regions and the re-initiation of transcription at each intergenic region is not 100% effective, a transcriptional gradient of mRNA from 3′ to 5′ is observed^26^. The relative genomic position of the ATU can thus be used to modulate the transcription rate, since when the ATU is closer to the 3′ end of the genome, higher amounts of mRNA are transcribed from the additional gene^28^.

Besides marker genes such as eGFP^29^, this opportunity was utilized early on to encode additional vaccine antigens, starting with hepatitis B virus small antigen HBsAg^30^. This review aims to describe the versatility of this platform technology. We would like to summarize different targets to generate MeV-derived vaccines against in the context of the nature of the respective diseases, and show the extent of the development, from the choice of MeV strains, the target antigens, and the animal models used for characterization, up to the considerable numbers of clinical trials.

### MeV-derived vectors targeting diseases acquired via the respiratory route

Diseases acquired via the respiratory route have been among the most intensively studied targets of live-attenuated MeV-derived vaccines. The strong attention attracted to this disease group stems from the high transmissibility of pathogens transmitted by droplets, aerosols, or dust, and their resulting potential for pandemic spread. Different viruses from the families of *Paramyxoviridae, Pneumoviridae, Arenaviridae, Orthomyxoviridae*, and *Coronaviridae* have been chosen as antigen donors (Table 1).

**Table 1:** Recombinant MeV-derived vaccines targeting diseases transmitted via the respiratory pathway.

Target	Antigen	ATU^a^	Strain^b^	IFNAR^−/−c^	CD46-mice^d^	Cotton rats	Syr. Hamsters^e^	Rhesus mac.	Cynomolgus	AGM^f^	HI Abs^g^	ELISA^h^	nAbs^i^	ELISpot^j^	ICS^k^	CTLs^l^	Challenge^m^	Clinical trial	Refs.
IAV	HA	P	Edm-Zagreb																^39^
	HA	P	AIK-C			X					X						X		^52^
	HA (H5)	N	Edm-B, HL_att_						X			X					X		^49^
LASV	NP + GPC Z + GPC	P pre-N + P	Schwarz						X			X	X^n^		X		X	I	^46,47^
MERS-CoV	S	P, H	Moraten		X							X	X	X		X	X		^33^
	S; N	H; P	Moraten		X								X	X	X				^42^
MuV	HN, F	P	Edm-B																^50^
	HN	P	Edm-Zagreb																^39^
NiV	G	N	Edm-B, wtHL				X			X		X					X		^48^
RSV	F	pre-N, P	Edm-Zagreb			X		X				X	X	X			X		^39,41^
	G, F	P	AIK-C			X			X				X				X		^51,53^
	G, F	Chimera^o^	AIK-C			X							X				X		^40^
	F, M2-1, NP	P	AIK-C			X						X	X				X		^44,45^
SARS-CoV	S, N	P	Edm-Zagreb		X							X	X	X					^43^
	S	P	Schwarz		X							X	X				X		^31^
SARS-CoV-2	S	H	Moraten		X		X					X	X	X	X	X	X	I/ II	^38^
	S	P	Schwarz	X	X	X	X					X	X	X	X		X		^32^
	S	P	Schwarz	X			X					X	X	X	X		X		^34^
	S	P^p^	Schwarz									X	X		neg^q^				^35,36^

Listed are all MeV-derived experimental vaccines that target diseases transmitted by the respiratory pathway. Described are the vaccine properties; depicted by “X” are the animal model(s) those have been tested in, positive immune responses detected in those models directed against the additional antigen(s), and efficacy in animal challenge models or clinical trials. Negative results of performed tests are labeled with neg. ^a^Genomic position of the additional transcription unit (ATU); pre-N indicates first position in the genome, N, P, H, or L indicate position of the ATU directly following N, P, H, or L gene cassettes, respectively. ^b^Vaccine strain, the backbone of respective recombinant MeV has been derived from. ^c–f^Preclinical or clinical model organism to analyze induction of immunity; ^c^ IFNAR^-/-^: mice with defect in innate Type I IFN responsiveness; ^d^ CD46-mice: Mice transgenic for MeV vaccine strain receptor CD46 and defect in innate Type I IFN responsiveness; ^e^Syr. hamsters: Syrian hamsters; ^f^AGM: African green monkeys. ^g^^–^^l^Antigen-specific immune responses triggered after immunization, which has been determined by measuring ^g^hemagglutination inhibiting antibodies (HI Abs), ^h^total binding antibodies (ELISA), ^i^neutralizing antibodies (nAbs), or reactive T cells determined by ^j^ELISpot or ^k^intracellular cytokine staining (ICS), as well as ^l^cell-mediated immunity via cytotoxic T lymphocytes (CTLs). ^m^Protective capacity of vaccine-induced immune responses after challenge of the appropriate animal model determined by reduction of pathogen load or attenuation of etiopathology. ^o^Vaccine virus with RSV F + G ectodomains fused to TM regions of MeV F + H in place of MeV F + H, respectively. ^n^nAbs against LASV after vaccination only in 1 out of 4 vaccinated animals, but enhanced nAb titers in all vaccinated animals after infection. ^p^ATU is not explicitly indicated, but referenced to viruses with SARS-CoV antigens in post-P position. ^q^In human vaccinees.

For the purpose of generating these vaccine candidates, mainly the surface proteins were used as antigen to be presented by the recombinant bivalent MeV. This choice is due to the potential of these proteins for the induction of neutralizing antibody (nAb) responses, which could protect against infection. All of the surface protein antigens were tested in their unmodified full-length form. For the vaccines targeting the coronaviruses SARS-CoV, MERS-CoV, or SARS-CoV-2, modified forms of the respective Spike proteins (S) were also tested to enhance their immunogenicity. For this purpose, genes encoding soluble S without membrane anchor^31–33^, or stabilized S frozen in the pre-fusion conformation by the introduction of few key mutations and deleting the protease cleavage motif separating the two subunits S1 and S2^32,34–36^ were generated and tested. Stabilization of S in the pre-fusion conformation enhances the presentation of portions of the protein that are targets of nAb^32,37^. One side effect of this is that these mutations reduce the hyperfusogenic phenotype of S-expressing MeV^38^, and stabilize antigen expression^34^.

As an alternative strategy to enhance immunogenicity of the encoded additional antigen, chimeric versions of the mumps virus (MuV) hemagglutinin-neuraminidase (HN) attachment protein^39^ or respiratory syncytial virus (RSV) F and G glycoproteins^40^ were generated. For these chimeric viruses, the cytoplasmic and transmembrane domain of the chimeric HN was derived from MeV H, or the RSV F and G ectodomains were substituted by the corresponding regions of MeV F and H, respectively. The latter changed the cell tropism of the recombinant vaccine to that of RSV^40^ demonstrating successful incorporation of the glycoproteins into MeV particles and generation of chimeric infectious particles. This generation of chimeric infectious vaccine virus is fundamentally different from the classical approach of co-expressing the foreign antigen, since the entry receptor tropism will be changed. Besides constituting a challenge for biosafety considerations, the effects of the change of tropism of the very lymphotropic MeV to other target cells has to be critically monitored also with a view to the balanced immunity MeV can induce. An alternative strategy is the expression of only the receptor-binding domain of SARS-CoV-2 S^32^ or the ectodomain of RSV F^41^ as soluble proteins to focus the immune reactions to critical regions of the antigens.

In addition to encoding the surface proteins, vaccine candidates were developed that target structural or regulatory proteins of the target pathogens, such as the nucleocapsid (N) proteins for MERS-CoV^42^, SARS-CoV^43^, RSV^44,45^, or Lassa virus (LASV)^46,47^, as well as the polymerase co-factor M2-1 of RSV^44,45^. The use of the conserved N proteins aims to induce broadly reactive T cell immunity to slow down the development of immune escape variants.

For some of the targets, vaccine candidates were developed in parallel that carried the antigen encoded in different ATUs, i.e. ATUs in the post-N, post-P, or the post-H position. High expression of a specific foreign antigen may interfere with replication of the recombinant vaccine virus. This is evident when unmodified MERS-CoV and SARS-CoV-2 S are used. If they are placed in the post-P ATU, the resulting vaccine viruses have significant growth defects^33,38^.

Hence, the optimal ATU for expression of the foreign antigen must be empirically determined for the respective antigen in combination with the MeV backbone utilized. For example, expression of Nipah virus (NiV) glycoprotein G expression from an ATU in the post-N position impaired growth of Edmonston B vaccine strain-derived backbone, while recombinant wt HL-derived virus grew normally despite encoding NiV-G in same position^48^. Vaccine viruses with influenza A virus (IAV) hemagglutinin (HA) derived from highly pathogenic avian strains^49^ or RSV F^41^ inserted in the post-N position were also successful. However, to ensure proper replication and antigen expression of vaccine viruses encoding MERS-CoV S or SARS-CoV-2 S, the additional genes had to be inserted further toward the 5′ proximal end of the genome, in the post-H ATU^33,38^. All other described vaccines representing the majority of candidates (12 out of 17) utilize the post-P ATU for expression, with few or no growth defects.

Interestingly, the diversity of the MeV strains used as backbones for the vaccine candidates against pathogens transmitted via the respiratory route was highest among all disease subgroups. Edmonston B^32,48–50^ or its derivatives Edmonston Zagreb^39,41,43^, Moraten^33,38,42^, Schwarz^31,34–36^ (that shares 100% nucleotide identity with Moraten), or the temperature-sensitive AIK-C^40,44,45,51–53^ vaccine strains were successfully tested. Moreover, even the wild-type HL strain^48^ or an attenuated clone of HL^49^, which was generated by genetically interfering with expression of the MeV accessory protein and virulence factor V^54^, were used to generate potential vaccines.

For testing of candidate vaccines, a diverse repertoire of animal models has been used. IFNAR^-/-^-CD46Ge mice have been the major animal model for testing these MeV-derived vaccines due to their permissiveness for the vector^31,33,38,42,43^. However, IFNAR^−/−^ mice without the CD46 transgene have also been used more recently^32,34^, and have been shown to host efficient replication of MeV independent of the hCD46 receptor transgene^55^. Alternatively, cotton rats are known to be semi-permissive for MeV, and were used to investigate immunogenicity and protection against RSV^39–41,44,45,51^, IAV^52^, or SARS-CoV-2^32^. Syrian hamsters turned out to be a good rodent model for COVID-19 pathogenesis and were predominantly used to analyze immune responses and protection of experimental vaccines against SARS-CoV-2^32,34,38^, having also been used successfully to demonstrate efficacy of the MeV-derived NiV vaccine^48^. Non-human primates are the only natural hosts of MeV other than humans and are not used as frequently as rodents, but vaccination of African green monkeys^48^, cynomolgus macaques^46,47,49,53^, and rhesus macaques^41^ have shown immunogenicity or efficacy for the MeV-vaccines against NiV^48^, IAV^49^, LASV^46,47^, or RSV^41,53^.

In these different animal models, binding antibodies were detected by ELISA after vaccination. For six out of nine target viruses, target-specific nAbs were induced, namely for RSV^39–41,45,51,53^, LASV^46^, MERS-CoV^33,42^, SARS-CoV^31,43^, or SARS-CoV-2^32,34–36,38^. These reached maximum neutralizing titers of up to 4000 PRNT_50_ for SARS-CoV-2^34^, 1000 IC_50_ for SARS-CoV^31^, and a VNT of 874 for MERS-CoV^33^. Results obtained by optimization of the coronavirus S antigen were more variable. Whereas a solubilized version of MERS-CoV S was found to induce slightly higher nAb titers than the full-length protein^33^, the opposite was observed for SARS-CoV^31^. Stabilizing the SARS-CoV-2 S protein in its pre-fusion conformation resulted in significantly higher nAb titers (up to 5.5-fold) than observed for native, full-length S protein^32^.

Cellular immune responses were also detected by ELISpot or intracellular cytokine staining (ICS) for five out of eight targeted viruses. Secretion of IFN-γ after re-stimulation with antigen protein or peptides was described for MeV-vaccines with antigens from LASV^46,47^, MERS-CoV^33,42^, RSV^41,44,45^, SARS-CoV^43^, or SARS-CoV-2^32,34,38^ and revealed a broad range of reactivity. Only 9 IFN-γ secreting cells/10^6^ splenocytes were found after re-stimulating vaccinated animal splenocytes with RSV-F^41^, whereas ~2500 IFN-γ secreting cells/10^6^ splenocytes were found for SARS-CoV-2 S^34^ or MERS-CoV S^33^. The T cell responses were further characterized for MeV-derived candidates targeting MERS-CoV S or SARS-CoV-2 S via ICS analysis for the expression of IFN-γ, TNF-α, or IL-2. Between 0.01–0.5% of CD4^+^ T cells and 0.02–3% of CD8^+^ T cells were found to secrete at least one of the cytokines after re-stimulation. Up to 75% of these reactive cells expressed more than one cytokine and thereby revealed to be multifunctional^32,34,38,42^.

Since these MeV vaccine candidates were highly immunogenic, protective efficacy was validated for seven out of eight target pathogens; follow-up studies based on the earlier mumps vaccine work have thus far not been performed. Survival of vaccinated animals after lethal challenges was demonstrated for the NiV vaccine in Syrian hamsters^48^ and the LASV vaccine in cynomolgus macaques^46,47^. Interestingly, the immunity that was induced by the LASV vaccine was almost sterilizing, as no infectious virus, and only low amounts of viral RNA, were recovered from vaccinated animals^46^. Notably, protection does not correlate with nAb induction, but rather T cell immunity directed against intracellular NP protein. Encouraged by these results, this vaccine candidate has been transferred into a clinical Phase I study (NCT04055454). For vaccines against IAV^52^ or RSV^39–41,45,51^ in cotton rats, highly pathogenic avian IAV^49^ or RSV^53^ in cynomolgus macaques, MERS-CoV^33^ or SARS-CoV^31^ in IFNAR^−/−^-CD46Ge mice, or SARS-CoV-2 in IFNAR^−/−^ mice^32,34^, IFNAR^−/−^-CD46Ge mice^38^, or in Syrian hamsters^32,34,38^, protection was demonstrated by reduced or undetectable histopathological changes, and the absence (or low levels) of infectious virus, viral proteins, or viral RNA in vaccinated animals. Regarding protection against SARS-CoV, the height of nAb titers correlated with the degree of protection during challenge^31^.

In conclusion, this group of vaccines targeting diseases acquired via the respiratory route showed promising results with respect to the induction of robust, long-term humoral and cellular immunity, as well as protective efficacy in relevant animal models. Further clinical studies would be beneficial so that their protective efficacy in human vaccinees can be further analyzed to advance their development and application.

### MeV-derived vectors targeting arthropod-borne diseases

Diseases transmitted by arthropod vectors are among the primary targets to fight emerging or re-emerging infections because of their zoonotic character with animal reservoirs among wildlife and the difficult control of arthropod vectors. With Crimean-Congo hemorrhagic fever (CCHF), Rift valley fever (RVF) and Zika virus (ZIKV), arboviral diseases are prominent among the list of blueprint priority diseases of the WHO^56^. Moreover, malaria transmitted by mosquitos is among the most deadly infectious diseases. Therefore, MeV-derived vaccines have been generated, which target six different pathogens transmitted by arthropods, as summarized in Table 2.

**Table 2:** Recombinant MeV vaccines targeting arthropod-borne diseases.

Target	Antigen	ATU^a^	Strain^b^	CD46-mice^c^	BL/6-hCD46^d^	AG-hCD46^e^	Cotton rats	SM^f^	Cynomolgus	ELISA^g^	nAbs^h^	ELISpot^i^	ICS^j^	Challenge^k^	Clinical trial	Reference
CHIKV	C-E3-E2-6K-E1	P	Schwarz	X					X	X	X	X		X	II	^57,64,91,93,97,98^
DENV	EDIII, EDIII-ectoM, Tetra-EDIII-ectoM	P	Schwarz	X						X	X		X^l^			^66,99^
	Tandem-EDIII	P	Moraten		X	X				X	X	X				^65,100^
	EDIII-HBsAg	N, P	Moraten	X							X					^85^
JEV	prM-E	P	AIK-C				X			X	X					^62^
*Plasmodium falciparum; P. berghei* ^m^ *(malaria)*	CS (*Pb* or *Pf*)	P	Schwarz	X						X		X	X	X		^59^
WNV	E	P	Schwarz	X				X		X	X			X		^63,101^
ZIKV	E	P	Schwarz	X						X	X	X		X	I	^67^
	prME, NS1,prME-NS1	pre-N, N N N, H	Edm-B	X						X X X	X X			X X		^102^

Listed are all MeV-derived experimental vaccines that target arthropod-borne diseases. Described are the vaccine properties; depicted by “X” are the animal model(s) those have been tested in, positive immune responses detected in those models directed against the additional antigen(s), and efficacy in animal challenge models or clinical trials. ^a^Genomic position of the additional transcription unit (ATU); N, P, H, or L indicate position of the ATU directly following N, P, H, or L gene cassettes, respectively. ^b^Virus strain, the backbone of respective recombinant MeV has been derived from ^c–f^ preclinical or clinical model organism to analyze induction of immunity; ^c^CD46-mice: Mice transgenic for MeV vaccine strain receptor CD46 and defect in innate Type I IFN responsiveness; ^d^BL/6-hCD46, C57/BL6 mice transgenic for huCD46; ^e^AG-hCD46, A129 mice transgenic for huCD46; ^f^SM: squirrel monkeys. ^g–j^ Antigen-specific immune responses triggered after immunization, which has been determined by measuring ^g^total binding antibodies (ELISA), ^h^neutralizing antibodies (nAbs), or reactive T cells determined by ^i^ELISpot or ^j^intracellular cytokine staining (ICS). ^k^Protective capacity of vaccine-induced immune responses after challenge of the appropriate animal model determined by reduction of pathogen load or attenuation of etiopathology. ^m^*Plasmodium berghei* used to model malaria in mice. ^l^Secretion of cytokines by stimulated primary human monocyte-derived dendritic cells.

Five different arboviruses, and one parasitic agent, were investigated as target for the development of live-attenuated MeV-based experimental vaccines: one alphavirus, Chikungunya virus (CHIKV); four flaviviruses, dengue virus (DENV), Japanese encephalititis virus (JEV), West Nile virus (WNV), and ZIKV; and the malaria parasite *Plasmodium falciparum*. For all arboviruses, the envelope proteins, the major targets for nAbs, were chosen as antigens to be expressed from the ATU in post-P position. This demonstrates the good compatibility of these antigens with the MeV vector backbone by allowing comparatively high rates of antigen expression in vaccine virus-infected host cells without significantly impairing the vaccine´s replication. For CHIKV, the envelope proteins were expressed in the context of all structural proteins to foster generation of CHIKV virus-like particles (VLPs) by vaccine-infected cells^57^, which adds to the immunogenicity of the vaccine^58^. While vaccine candidates targeting WNV only encoded a soluble, C-terminally truncated version of E, vaccines against DENV, JEV, and ZIKV were generated that co-expressed the flaviviral precursor membrane chaperone protein prM, which is a second structural antigen, but also aids the proper expression of E. For vaccination against *Plasmodium falciparum* or *Plasmodium berghei*, the latter to be able to perform a malaria challenge model in mice, Mura et al.^59^ choose the circumsporozoïte protein (CS) as the target antigen, similar to the RTS,S/AS01 adjuvanted protein vaccine candidate, which has advanced to phase III clinical trials^60^.

Most of the vectors targeting arthropod-borne diseases have been originally developed in the laboratory of Frédéric Tangy, Institut Pasteur and utilize the Schwarz strain backbone^61^. Only JEV or some DENV vaccine candidates use the AIK-C or the Moraten strain backbones, respectively. In any case, all of these candidates have proven to be considerably immunogenic. Again, the IFNAR^−/−^-CD46Ge mouse model was used as the common standard of testing for all but the JEV E-encoding vaccine, which was tested in cotton rats^62^. However, mouse data were confirmed in squirrel monkeys or other non-human primate models for the WNV^63^ and the CHIKV vaccines^64^, respectively. For the DENV vaccine, A129 mice were additionally used to demonstrate efficacy^65^.

All vaccine candidates induced humoral responses in the respective animal models, since antibodies binding the additional target antigen were detectable along with the anti-vector responses. Moreover, these antibodies were also neutralizing. Titers were in the range of 300 PRNT_50_ for the vaccines targeting DENV^66^, JEV^62^, WNV^63^, and ZIKV^67^ in IFNAR^−/−^-CD46Ge mice or cotton rats. An exception was the vaccine targeting CHIKV, with a PRNT_50_ of ~10^4^ after prime-boost vaccination^57^. Robust antigen-specific T cell responses were also described for the vaccines encoding additional antigens of CHIKV^57^, ZIKV^67^, or *Plasmodium*^59^. The numbers of T cells against the antigen of choice were in the range of 150–200 IFN-γ reactive Ag-specific T cells/10^6^ splenocytes. These numbers were too low to properly assess multi-functionality of the respective T cells.

These significant immune responses, albeit not as strong as MeV vaccines targeted against respiratory pathogens, demonstrated to be protective in challenge experiments for all but the JEV and DENV vaccine candidate, which thus far has not been tested. In mouse studies, vaccination against ZIKV was not only protective for the vaccinated dam, but also for its unborn offspring against infections during pregnancy^67^. Consequently, two of the vaccine candidates were brought into clinical development. While the ZIKV vaccine entered two phase I clinical trials (Table 3), the CHIKV vaccine successfully completed phase II clinical trials^68^ (Table 3), and would be ready to enter phase III trials to prepare the first marketing authorization of a vaccine utilizing the live-attenuated measles virus platform.

**Table 3:** Clincial Trials testing Live-attenuated MeV-derived vaccines.

Trial number	Virus	Disease	Phase	Institution	Status	Refs.
EudraCT 2013-001084-23	MV-CHIK	Chikungunya fever	I	Themis Biosciences	Completed	^93^
NCT01320176	MV1-F4-CT1	AIDS	I	Institut Pasteur	Completed	
NCT02861586	MV-CHIK	Chikungunya fever	II	Themis Biosciences	Completed	^91,98^
NCT02996890	MV-ZIKA	Zika fever	I	Themis Biosciences	Completed	
NCT03028441	MV-CHIK	Chikungunya fever	I	NIAID	Completed	
NCT03101111	MV-CHIK	Chikungunya fever	II	Themis Biosciences, Walter Reed Army Institute of Research	Completed, results posted	
NCT03635086	MV-CHIK	Chikungunya fever	II	Themis Biosciences	Completed, results posted	
NCT03807843	MV-CHIK	Chikungunya fever	II	Themis Biosciences, Walter Reed Army Institute of Research	Completed, results posted	
NCT04033068	MV-ZIKA-RSP	Zika fever	I	Themis Biosciences	Completed, results posted	
NCT04055454	MV-LASV	Lassa fever	I	Themis Biosciences	Completed	
NCT04497298	TMV-083 / V-591	COVID-19	I	Institut Pasteur, Themis Biosciemces, CEPI	Completed	^35^
NCT04498247	V591	COVID-19	I / II	Merck Sharp & Dohme	Terminated	^36^

Listed are clinical trials testing recombinant MeV-derived vaccines as identified in public databases with increasing clinical trial designation number.

### MeV-derived vectors targeting diseases transmitted by fluids or sexual contact

As already mentioned, the first attempts to generate an effective bivalent vaccine derived from MeV targeted a pathogen transmitted by direct contact, hepatitis B virus (HBV)^30^, and was envisioned as an effective, inexpensive alternative to the authorized, but relatively expensive, VLP-type vaccines. To date, a variety of other pathogens transmitted by direct contact have been targeted, not least because of the inherently long-lived, strong humoral and cellular immune responses triggered by the application of the MeV vaccine platform (Table 4).

**Table 4:** Recombinant MeV vaccines targeting diseases transmitted by fluids or sexual contact.

Target	Antigen	ATU^a^	Strain^b^	IFNAR^−/− c^	CD46-mice^d^	hum. Mice^e^	Cotton rats	Rhesus mac.	Cynomolgus	ELISA^f^	nAbs^g^	ELISpot^h^	ICS^i^	Challenge^j^	Clinical trial	Refs.
EBV	gB350	N, P	Edm-Zagreb				X	X		X	neg	X				^41^
HBV	HBsAg	P	Edm-B		X					X						^30^
	HBsAg	N, P, H, L	Moraten		X			X		X				MeV		^28,69^
HCV	E1, E2	N	Edm-B			X				X						^70^
	C, E1, E2; E1/Ft, E2/Ft	P	Moraten		X					X	X					^71^
*Helicobacter pylori*	NAP	pre-N	Edm-B	X	X					X		X				^84^
HIV-1	Env	P, H	Edm-B		X			X		X	X	X	X		I	^82^
	Env	P	Schwarz		X					X	X		X			^83^
	Gag + Env	P H	Schwarz		X					X	X	X				^76^
	Env, Gag + Pol; Gag	P P, H; P	Moraten		X					X		X				^75^
	F4	P	Schwarz		X				X	X			X			^79–81^
HPV	L1	P	Edm-Zagreb		X			X		X	X					^72,73^
SHIV	Gag, Env; Nef	P, H; pre-N	Schwarz						X	X	X	X	X	X		^78^
SIVmac	Env; Pol; Gag	P, H; P; H	Edm-B													^50^
	Env (+ Pol); Gag	P; H	Edm-B		X					X						^74^
	Gag	P	Edm-Zagreb					X		X		X	X			^77^

Listed are all MeV-derived experimental vaccines that target diseases transmitted by fluids or sexual contact. Described are the vaccine properties; depicted by “X” are the animal model(s) those have been tested in, positive immune responses detected in those models directed against the additional antigen(s), and efficacy in animal challenge models or clinical trials. Negative results in performed assays are labeled with neg. ^a^Genomic position of the additional transcription unit (ATU); pre-N indicates first position in the genome, N, P, H, or L indicate position of the ATU directly following N, P, H, or L gene cassettes, respectively. ^b^Vaccine strain, the backbone of respective recombinant MeV has been derived from. ^c–e^Preclinical or clinical model organism to analyze induction of immunity; ^c^IFNAR^−/−^: mice with defect in innate Type I IFN responsiveness; ^d^CD46-mice: Mice transgenic for MeV vaccine strain receptor CD46 and defect in innate Type I IFN responsiveness; ^e^hum. mice: humanized mice - NOD/Scid/Jak3null mice engrafted with human peripheral blood leukocytes (hu-PBL-NOJ). ^f–i^Antigen-specific immune responses triggered after immunization, which has been determined by ^f^measuring total antibodies (ELISA), ^g^neutralizing antibodies (nAbs), or reactive T cells determined by ^h^ELISpot or ^i^intracellular cytokine staining (ICS). ^j^Protective capacity of vaccine-induced immune responses after challenge of the appropriate animal model determined by reduction of pathogen load or attenuation of etiopathology.

Besides significant effort on vaccines against human immunodeficiency virus (HIV-1) and related simian or hybrid immunodeficiency viruses (SIV or SHIV, respectively) that allow to use non-human primate models for immunodeficiency virus challenge, recombinant MeV encoding foreign antigens from Epstein-Barr virus (EBV)^41^, HBV^28,30,69^, hepatitis C virus (HCV)^70,71^, human papilloma virus (HPV) high-risk serotopyes HPV16 and HPV18^72,73^, or the bacterium *Helicobacter pylori* have been generated. All of these recombinant vaccine candidates triggered significant antibody responses in immunized animals.

The major target antigens for all of these different pathogens are their surface proteins: MeV-derived vaccine candidates against EBV, HBV, HCV, and HPV exclusively rely on the respective surface proteins. Vaccines against HIV-1 and related viruses (SIV or SHIV) also utilize the group-specific antigen (Gag)^50,74–78^ or a fusion protein composed of HIV-1 matrix protein p17, capsid protein p24, reverse transcriptase and Nef (F4)^79–81^. Moreover, HIV-1 envelope protein (Env) was modified to be used as an optimized antigen in the MeV context. Membrane-anchored or secreted variants of Env were tested in the native sequence^82^ or with deletions of certain variable loops to enhance broad immunogenicity and foster development of functional antibody responses by de-targeting those from highly flexible target structures^82,83^. EBV gB350 surface protein was also cloned in a soluble form into MeV^41^, while HCV E1 and E2 were either fused with the cytoplasmic tail of MeV fusion protein to enhance incorporation into and presentation by MeV particles or expressed as a heterodimer^71^. For the other target viruses, the surface proteins were used without modifications.

In addition to the binding antibodies detected by ELISA for all experimental vaccines, four of the eight vaccine candidates induced nAbs, namely those encoding the antigens of HCV^71^, HIV-1^76,82,83^, SHIV^78^, and HPV^72,73^. Target-specific nAb titers varied considerably. The range spans from an IC_50_ of 30 for SHIV^78^ up to ~63,000 for HPV^73^. For the HBV-vaccines^28,30,69^, only the neutralization capacity of sera against the MeV vector backbone were verified.

Pathogen-specific T cell responses were characterized predominantly by ELISpot, but also ICS and FluoroSpot analysis, and induced by all five vaccine candidates that were analyzed. MeV-derived vaccines targeting EBV^41^, HIV-1^75,76,80–83^, SHIV^78^, SIV^77^, or *Helicobacter pylori*^84^ induced both vector- and target-specific T cell responses. Only MeV-specific T cell responses were characterized for the vaccines targeting HBV^28,69^. The target-specific cellular immune responses detected via IFN-γ ELISpot ranged from barely-detectable 23 spots /10^6^ splenocytes for the anti-EBV vaccine^41^ up to 1200 spots /10^6^ splenocytes for the HIV-1 vaccine expressing a modified Env protein on VLPs derived from Gag^76^. Moreover, multifunctional T cells expressing IFN-γ, IL-2, or TNF-α were induced by MeV expressing the F4 antigen of HIV-1^80,81^. About 11% of CD4^+^ T cells secreted IFN-γ upon recall, but only 0.09% also stained positive for IL-2^80^.

For these combinations of diseases and respective vaccines, IFNAR^−/−^-CD46Ge mice were the main model used for experiments. Remarkably, for six out of eight vaccine candidates, non-human primate models were used to confirm the vaccine candidates´ immunogenicity. Cotton rats were used to test MeV-derived EBV vaccine candidates^41^, which revealed the induction of target antigen-specific antibody and T cell responses. In contrast, a humanized mouse model (hu-PBL-NOJ), failed to display any immune responses^70^. Despite the demonstration of strong in vivo immunogenicity by all candidates, protective efficacy was recently confirmed only for one vaccine by challenge of cynomolgous macaques with SHIV-SF152p3 after vaccination with a Schwarz strain-derived MeV encoding the HIV-1 Gag, Env, and Nef antigens^78^. The lack of animal models that are not only permissive to MeV, but also reproduce infection of human diseases may explain the scarcity of protective efficacy studies for these diseases. Nevertheless, one MeV-derived vaccine candidate encoding antigens of HIV-1 was brought into the clinic and tested in a phase I clinical trial (NCT01320176), but the results of the study have yet to be published.

While this group of vaccines is not as advanced in studies proving its protective efficacy in animal models or the clinic, early studies have greatly enhanced our understanding of the MeV vaccine platform. By testing MeV expressing HBsAg of HBV from ATUs positioned at four different sites of the MeV genome (post-N, post-P, post-H, and post-L), the impact of the amount of antigen produced on the immunogenicity of the recombinant vaccines due to the transcriptional gradient of MeV could be studied, as outlined in the introductory section of this review. MeV expressing HBsAg in post-P, post-H, or post-L positions revealed considerably different HBsAg-specific antibody titers correlating with amounts of expressed antigen^28^. However, although expression of HBsAg was highest if encoded in the post-N position, post-P constructs induced anti-HBsAg titers in the same range^69^.

Moreover, the use of HBV demonstrated the beneficial effects of VLPs for immune-stimulation in the context of MeV vaccines, which was also described for MV-CHIK. When HBsAg was modified to display domains of DENV E protein, this hybrid antigen gave rise to VLPs that induced robust DENV-nAb responses in mice, while recombinant MeV encoding only the DENV E domains did not trigger anti-DENV nAbs^85^. While antibodies against the particular DENV domain have the potential to be highly neutralizing and protective, its small molecular size requires formation of subviral particles to be immunogenic^86^. Co-expression of HIV-1 Gag in addition to Env also proved to be strongly immunogenic in mice, correlating with VLP-formation^76^.

Furthermore, the beneficial effect of boosting with a low dose of adjuvanted protein antigen for the respective immune responses could be demonstrated by analyzing MeV targeting HBV and HCV among this group of vaccine-candidates employing such an immunization strategy^69,71^. In addition to viral pathogens, MeV is also an excellent vector platform for the presentation of bacterial antigens as shown for *Helicobacter pylori* by expression of secreted neutrophil-activating protein (NAP)^84^.

Taken together, this group of vaccines shows promising results regarding strong and long-lasting induction of pathogen-specific humoral and cellular immune responses. However, demonstration of proof-of-concept for efficacy in in animal models needs to be prioritized to foster clinical studies analyzing these promising MeV-derived vaccine candidates.

### MeV-derived vectors targeting cancer

Finally, recombinant MeV has not only been tested against transmissible diseases. Vaccine strain-derived MeV has also been actively developed as a so-called oncolytic virus for cancer therapy, which is reviewed elsewhere^87^. While these and other viruses were originally developed as anti-tumoral agents due to direct tumor cell killing by virus infection and replication, oncolytic viruses turned out to have an additional immunotherapeutic mode of action^88^. Most approaches with oncolytic MeV that aim to take advantage of the stimulation of immune cells against infected tumor cells try to enhance immunotherapeutic efficacy, either by additionally encoding stimulatory cytokines, or by relieving the immunosuppressive microenvironment in tumors. Nevertheless, two MeV-derived viruses have been described that encode additional selected tumor cell antigens to induce directed anti-tumoral immune responses as a tumor vaccine (Table 5).

**Table 5:** Recombinant MeV-derived cancer vaccines.

Target	Antigen	ATU^a^	Strain^b^	CD46-mice^c^	ELISA^d^	FACS^e^	CDC^f^	ELISpot^g^	Cytokines^h^	Efficacy^i^	Clinical trial	Refs.
Carcinomas (melanoma)	CLDN6	P	Moraten	X		X	X	X		X		^89^
*Helicobacter pylori*	HspA (α-tumor)	pre-N	Edm-B	X	X			X				^103^
>85% human cancers	TERT	P	Schwarz	X	neg			X	X			^90^

Listed are all MeV-derived experimental vaccines that target cancer. Described are the vaccine properties; depicted by “X” is the animal model those have been tested in, positive immune responses detected in those models directed against the additional antigen, and efficacy in animal challenge models or clinical trials. ^a^Genomic position of the additional transcription unit (ATU); pre-N indicates first position in the genome, N, P, H, or L indicate position of the ATU directly following N, P, H, or L gene cassettes, respectively. ^b^Virus strain, the backbone of respective recombinant MeV has been derived from ^c^ CD46-mice: mice transgenic for MeV vaccine strain receptor CD46 and defect in innate Type I IFN responsiveness. ^d–h^Antigen-specific immune responses triggered after immunization, which has been determined by measuring total binding antibodies (^d^ELISA or ^e^FACS), functional antibodies (^f^complement-dependent cytotoxicity, CDC), or reactive T cells determined by ^g^ELISpot or ^h^cytokine secretion of re-stimulated splenocytes. ^i^Anti-tumoral efficacy of vaccine-induced immune responses after challenge or treatment of the appropriate tumor model determined by reduction of tumor load or number of metastases or prolongation of survival.

The choice of the tumor antigens, claudin-6 (CLDN-6, an occludin representing an onco-fetal antigen) and telomerase reverse transcriptase (TERT, up-regulated in a broad range of human tumors) both reflect targeting of a broad range of tumors, since these antigens are overexpressed during oncogenic transformation in a wide range of cancers. Of note, the murine homolog of CLDN-6 was used to completely simulate the situation of central immune tolerance against an autoantigen in a mouse model^89^. Both antigens were tested in modified set-ups to enhance the immunogenicity. hTERT was encoded as a ubiquitin-fusion protein^90^, whereas an alternative MeV-derived CLDN-6 vaccine, MV_vac2_-gag-CLDN6, was additionally encoding retroviral Gag protein, that gives rise to the release of CLDN-6-presenting VLPs from vaccine-infected cells^89^.

Those tumor antigens were expressed from the post-P ATU, demonstrating compatibility of co-expression also of tumor antigens with MeV biology. This is also noteworthy for the virus co-expressing CLDN-6 and retroviral Gag, as the additional *gag* was cloned into a pre-N ATU and is highly expressed, but the resulting vaccine was genetically stable and showed unimpaired replication^89^. Thus, recombinant MeV can provide simultaneous high expression of two antigen moieties if these proteins do not interfere with replication of the MeV vector backbone. The highly comparable recombinant genomic backbones derived from the Schwarz or Moraten strain have been used as major platforms to generate the candidate vaccines against infectious diseases. Also the animal model for assessment of induced immune responses and for the CLDN-6 vaccines also prophylactic and therapeutic efficacy was similar, since in both studies, IFNAR^−/−^-CD46Ge mice were used.

While the MeV encoding hTERT only induced TERT-specific T cell responses and was significantly enhanced by priming with DNA vaccines^90^, the MeV encoding VLP-presented muCLDN-6 induced both CLDN-6-specific T cells as well as antibodies binding to CLDN-6, which were capable of inducing complement-dependent cytotoxicity^89^. Interestingly, MeV encoding only CLDN-6 induced humoral and cellular antigen-specific immune responses, thereby demonstrating the high immunogenicity of the MeV vector platform and its capability of breaking immune tolerance in a situation of antigenic homology. For the CLDN-6 vaccines, this remarkable immunogenicity translated into prophylactic and therapeutic efficacy in models of metastatic or cutaneous melanoma, respectively^89^. Thus, while there are fewer MeV-based vaccines in this group, the results achieved with tumor vaccines are among the most impressive in demonstrating the high immune-stimulatory capacity of live-attenuated MeV.

### Current state and future challenges

**Fig. 2:**
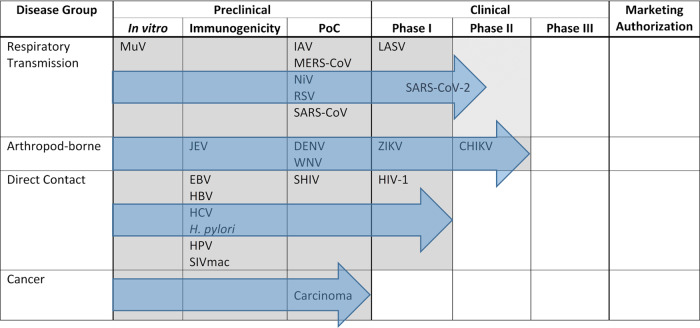
Progression diagram of current state of MeV-derived vaccine development. Depiction of progress of MeV-derived vaccines´ development targeting pathogens from groups of diseases differentiated by their mode of transmission. Outlined are the different preclinical and clinical developmental stages of vaccine development until marketing authorization. Position of the pathogens indicate most advance development of a vaccine candidate against this pathogen. Gray areas and blue arrows depict progress of the most advanced vaccine candidate directed against one pathogen out of the respective disease group. PoC, proof of concept in animal challenge experiment.

In the preceding sections, we have tried to give an overview of the versatility of live-attenuated MeV as a platform to generate vaccines against diseases transmitted by the respiratory route, direct contact, or arthropod vectors, as well as against cancers. However, the developmental progress of the respective vaccine candidates is quite variable. The progress of these platforms is summarized in a progression diagram (Fig. 2), which shows the most advanced stage of development reached by MeV-derived vaccines targeting the respective pathogens.

Interestingly, progress of development can be differentiated for the target categories. While proof of efficacy has been demonstrated for only one of the experimental vaccine candidates targeting a disease transmitted by direct contact, MV-SHIV^78^, all but one of the experimental vaccines targeting diseases transmitted via the respiratory route or arthropod vectors have yielded evidence of protection in animal models. For the respiratory group, the COVID-19 vaccine candidate has entered clinical development^35,36^, as has the LASV vaccine candidate (Table 3). More impressively, the most clinically advanced group is those vaccines targeting arboviral pathogens, with the MV-ZIKA vaccine having undergone testing in phase I (NCT02996890, NCT04033068), and MV-CHIK having succeeded in phase II clinical trials^91^ ready to enter phase III (Table 3 and Fig. 2). If these trials are successful, marketing authorization could be expected. Interest of key players of the pharmaceutical industry in this technology became evident at least when Merck Sharp & Dohme acquired Vienna-based Themis Biosciences^92^, who have been driving clinical development of the MeV-derived vaccines against CHIKV^68,93^, ZIKV (NCT02996890, NCT04033068), and SARS-CoV-2^35,36^. Moreover, the very first project funded by the Coalition for Epidemic Preparedness Innovations (CEPI) focused on the development of MeV-derived vaccines against LASV and MERS-CoV^94^ and financed the development of the LASV vaccine into clinical evaluation (Table 3, NCT04055454).

**Fig. 3:**
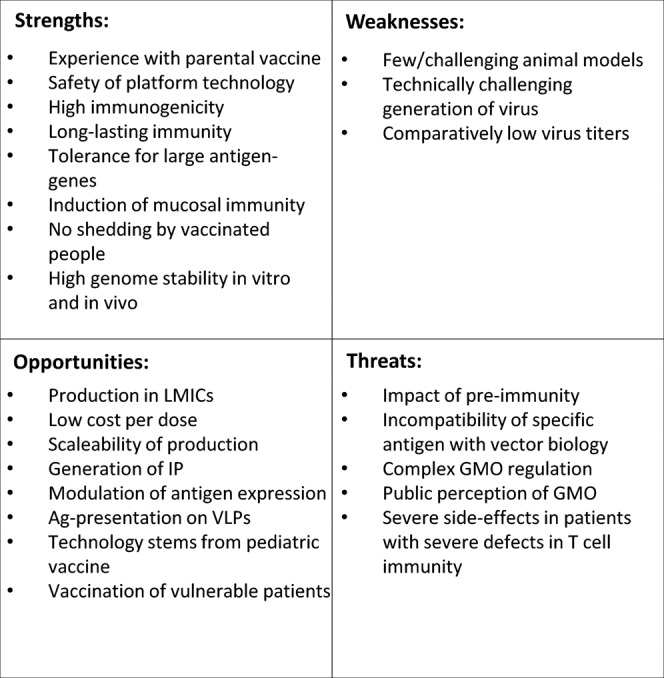
SWOT analysis of live-attenuated MeV as vaccine platform technology. Depicted are strength, weaknesses, opportunities and threats of the use of live-attenuated recombinant MeV as platform technology to generate vaccines against other pathogens. LMICs low- and middle-income countries, IP intellectual property, Ag antigen, VLPs virus-like particles, GMO genetically modified organism.

While these are promising aspects, there are undoubtedly some challenges and drawbacks of this technology, as summarized by SWOT analysis (Fig. 3). Most prominently discussed is the impact of measles pre-immunity in potential recipients. For other vector systems, especially vaccine vectors derived from serotype 5 adenoviruses (Ad5), serotype-specific pre-immunity has been assigned as detrimental to vaccination success. During the phase III STEP trial testing an Ad5-vectored vaccine against HIV-1^95^ it was found that the vaccine did not protect subjects with a pre-formed anti-Ad5 serum titer, but instead enhanced the risk of HIV infection in this cohort. These findings were related to activation of the dendritic cell – T cell axis by vector-immune complexes facilitating entry of HIV-1 into its thereby activated target cells^96^. However, for MeV-derived vectored vaccines, animal models demonstrated the capacity to trigger at least humoral responses against the target antigen in mice^57,82^ and non-human primates^82^ for both HIV-1- and CHIKV-vaccine candidates, and were in accordance with early clinical trial data of the MV-CHIK vaccine^93^. These trials demonstrated similar seroconversion of patients to the target antigen independent of their anti-MeV serum status prior to the trial. However, the picture became different for the MeV-vectors targeting SARS-CoV-2. Here, pre-formed anti-measles immunity negatively correlated with anti-COVID-19 responses^35^. This may indicate that the impact of anti-measles immunity on clinical efficacy of MeV-derived vaccine vectors is dependent of the antigen (native CHIKV-E vs. stabilized SARS-CoV-2 S), the way the antigen is presented by the vaccine (VLPs vs. cell-associated), or some other parameters yet to be defined. In any case, deeper understanding of the tight interactions of MeV with the immune system, which is a direct virus-host relation, will be helpful in solving this enigma, also in comparison to other vector systems. Taking advantage of these processes could also be helpful to tailor future vaccine candidates to overcome these challenges and to further improve efficacies.

## Conclusion

Vaccine candidates that utilize live-attenuated MeV as a platform directed against 22 pathogens representing diseases transmitted via fluids or sexual contact, insect vectors, or the respiratory route have been described. These, as well as three experimental cancer vaccines, have shown induction of robust humoral and cellular immunity and often impressive efficacy in animal models of disease. This is even more remarkable, since animal models to test MeV-derived vaccines against a given disease have not only to be susceptible to the respective pathogen and to reflect the cause of disease, but they must also respond to the MeV-derived vaccine. With the parental MeV naturally showing strict primate tropism, these are notoriously difficult to establish. These data led to the realization of at least 11 clinical trials, all demonstrating the expected high safety profile. Moreover, four of those trials have tested the Chikungunya vaccine in a phase II clinical trial that showed evidence of efficacy in humans. Therefore, this platform technology is on the cusp of being transformed from an experimental concept into real-world relevance. The recent outcomes of the respective MeV-derived COVID-19 vaccine trials with non-competitive immunogenicity and indications of detrimental effects of measles pre-immunity have been somewhat sobering in this respect. Nevertheless, the accumulated data revealed significant impacts of the specific antigens, how the antigens are presented (incorporated vs. presented on VLPs), and the MeV strain which was used as the backbone for the experimental vaccine, on the vaccines´ efficacy. Thus, there seems to be ample room for optimization of this promising vector platform and its application. A better understanding of the interactions of the immune system with this highly lymphotropic, live-attenuated vaccine virus in combination with a given antigen, and accumulating experience in further clinical trials will pave the way for future successful development.

## Data Availability

Data sharing not applicable to this article as no datasets were generated or analyzed during the current study.
